# Microbiome and metabolomic changes in rabbits induced by *Folium sennae*

**DOI:** 10.1371/journal.pone.0320496

**Published:** 2025-03-31

**Authors:** Houkang Ding, Ming Li, Ning Ma, Shahid Ali Rajput, Mikhlid H. Almutairi, Bader O. Almutairi, Zhaoqing Han, Aituan Ma, Dengshan Shiau

**Affiliations:** 1 Department of Traditional Chinese Veterinary Medicine, College of Veterinary Medicine, Hebei Agricultural University, Baoding, Hebei, China; 2 Department of Animal Engineering, Yancheng Bioengineering Branch, Jiangsu Union Technical Institute, Yancheng, Jiangsu, China; 3 Department of Animal and Dairy Sciences, Faculty of Veterinary and Animal Sciences, Muhammad Nawaz Shareef University of Agriculture, Multan, Pakistan; 4 Zoology Department, College of Science, King Saud University, Riyadh, Saudi Arabia; 5 College of Agriculture and Forestry, Linyi University, Linyi, Shandong, PR China; 6 Department of Graduate Studies, Chi University, Reddick, Florida, United States of America; Zagazig University Faculty of Agriculture, EGYPT

## Abstract

Diarrhea is a serious health concern in livestock, affecting productivity and animal welfare. However, it remains a significant threat. Various practical therapies are being explored, such as prebiotics, probiotics, and organic acids, as well as chemical treatments including antibiotics, to combat this disorder. This study aims to explore the microbiome and metabolomic changes in rabbits with diarrhea. In the present study, diarrhea was induced in rabbits via oral gavage of *Folium Sennae* (FSAE), to assess body weights, diarrhea index, serum biochemical indicators, histopathology, microbiota, and metabolomics changes. Our study provides new insights into the mechanism of FSAE-induced diarrhea in rabbits and offers a novel mechanism for the interaction between gut microbiota, metabolomics, and gastrointestinal (GIT) dysfunction. Our results revealed that rabbits receiving FSAE showed a markedly higher diarrhea index and reduced body weight. Notably, levels of somatostatin, substance P, cholecystokinin, 5-hydroxytryptamine, vasoactive intestinal peptide, and acetylcholinesterase were significantly increased compared to control (P <  0.01). However, the levels of gastrin, motilin, enkephalin, and β-Endorphins were significantly decreased (P <  0.01). Microbial analysis revealed a significant reduction in microbial diversity (Shannon, Simpson, ACE, and chao1) and a decrease in Firmicutes*,* Campilobacterota*,* and Proteobacteria populations in FSAE-exposed rabbits. Additionally, 13 key metabolites associated with taurine and hypotaurine metabolism, alanine, aspartate and glutamate metabolism, starch and sucrose metabolism, histidine metabolism, and citrate cycle were identified in the colonic tissues. The present study concludes that FSAE-induced diarrhea in rabbits is associated with significant histopathological alterations in the colon, dysregulation of serum biochemical markers, and dysbiosis in metabolomics and gut microbiota. Our findings offer a novel model for investigating GIT dysfunction and its potential treatments.

## Introduction

Diarrhea refers to the higher frequency of bowel movements than normal, thin feces, increased water content, and the presence of undigested food with pus. The high incidence rate of diarrhea is an important cause of animal mortality [1–3]. Animal models of diarrhea are essential for exploring the treatment of this disease. Several methods have been used to induce diarrhea in animals, e.g., bacteria (such as *Escherichia coli*, and *Salmonella*), viruses (norovirus and rotavirus), or parasites (*Cryptosporidium*) are used for establishing an infectious diarrhea model [4–6]. Chemicals such as magnesium sulfate, fructose, lactose, and mycotoxins have been administered to generate diarrhea in animals [7,8]. However, some limitations of these methods include administration time, dosage, individual differences, and difficulties in administration have been reported.

*Folium Sennae* (FSAE) is often used in animal studies to establish diarrhea [9]. The main active ingredients of FSAE include anthraquinones such as sennaoside A and sennaoside B [10]. In rats, diarrhea was successfully established by using a FSAE for 4 weeks in rats at the dosage of 10 mL/kg by gavage [11]. Gut microbiota performs complex metabolic activities within the intestine and directly influences the gut environment [12]. Animals suffering from diarrhea have been reported to have fewer beneficial bacteria and more pathogenic bacteria in the gut compared to healthy animals. In an animal model of diarrhea induced by rhubarb leaves, it was observed that there was a reduction in colonic microbial diversity along with changes in the composition of species belonging to Bacteroidetes and Firmicutes phyla, Paraprevotella, Streptococcus and Epulopiscium [13]. Dysbiosis of gut microbiota disrupts intestinal mucosa barriers, leading to alterations in host metabolic pathways. Researchers have applied network pharmacology and KEGG enrichment to analyze microbiota and found alterations in tryptophan products induced by rhubarb leaves [14]. Therefore, analysis of gut microbiota in the diarrhea model helps to explore microbiota changes and provides potential insights for its treatment.

Metabolomics studies indicate the overall composition and changes of metabolites [15,16]. In diarrheal studies, metabolomics can identify metabolic abnormalities by analyzing metabolites in urine, blood, tissue, or intestinal contents. Metabolomic analysis of feces has revealed significantly lower total short-chain fat acids in animals suffering from diarrhea compared to normal animals. As mentioned earlier, the condition harms intestinal microarchitecture and electrolyte balance [17,18]. Additionally, it has been observed that lipid and bile acid metabolism disorders in fecal metabolites of rats with diarrhea involve disruptions in metabolisms of medium or long-chain fatty acids and bile acid [12,19].

As limited reports are present on the metabolic changes in the intestine caused by diarrhea, therefore, this study offers a unique perspective by combining both microbiota and metabolomics approaches. In the present study, diarrhea was induced in rabbits by FSAE, and changes in body weight, diarrhea index, behavior, pathology, biochemical index, microbiota, and metabolomics were performed to explore the potential mechanisms of diarrhea.

## Materials and methods

### Folium Sennae

*Folium Sennae* was prepared following a previously published method [9] with minor modifications. Briefly, FSAE was purchased from Tongrentang pharmacy (Beijing, China) and then soaked with 1 L of water in a beaker for 30 min, after which it was placed into a water bath that was heated to 100℃. These conditions were maintained for 25 min, and filtrate was obtained. This procedure was repeated 20 times to produce 12 L of FSAE. The obtained solution was condensed by rotary evaporator to 2 L, equal to 1 g/mL of FSAE, and then stored at 4 ℃.

### Ethics statement

The experimental procedures were reviewed and approved by the Laboratory Animal Ethics Committee of Hebei Agricultural University (Approval No. 2021066).

### Experimental animals

Twelve male New Zealand rabbits aged 5 months weighing 2.0 ~ 2.5 kg were purchased from The Quanyou Farm (Baoding, China) and fed at the Hebei Agricultural University animal facility. Each rabbit was kept in a cage with a temperature of 22 ±  3°C 50% ~  60% relative humidity and free access to water and feed. After acclimatization for seven days, the animals were grouped into the control group (n = 6) and the FSAE group (n =  6). The random number of groupings was generated by the Random Function in Microsoft Excel. In the control group, normal saline was administrated by gavage at a dosage of 8 mL/kg daily for two weeks, and the same dosage of FSAE was given to the animals in the model group simultaneously. The gavage was administered daily at 10 am, and the procedures were completed within half an hour. To ensure the efficacy of FSAE, no other medicine was used during the experiment [20]. Body weight was recorded every week, and the diarrhea index (percent of loose stools ×  diarrhea degree) was calculated to evaluate the condition of rabbits [21]. Data from rabbits that experienced an infection or premature death was excluded from the study. At the end of the trial, all rabbits were euthanized by the pentobarbital sodium method (100 mg/kg). Blood samples were collected from jugular veins, and serum was separated by centrifugation for ELISA. Moreover, the colonic content and tissue were taken for gut flora metabolomics and histopathological analysis.

### ELISA and pathology analysis

The levels of gastrin (GAS), motilin (MLT), somatostatin (SS), Substance P (SP), vasoactive intestinal peptide (VIP), cholecystokinin (CCK), 5-hydroxytryptamine (5-HT), acetylcholinesterase (ACHE), enkephalin (ENK), β-endorphins (β-EP) in serum were analyzed by ELISA kits from Enzyme-linked Biotechnology (Shanghai, China). The colon tissues were dissected, fixed, paraffin-embedded, and stained with hematoxylin and eosin. A simple compound microscope (Olympus, Japan) was used for histopathological examination.

### Microbiota sequencing in colonic content

The DNA from colonic content was extracted using the Magen HiPure Stool DNA kit (Guangzhou, China), and 341F/806R primer was used for 16S rRNA gene amplification. Then, PCR products were purified, quantified, and sequenced on the Illumina NovaSeq PE250 platform (San Diego, USA).

Raw reads were merged via FLASH, and quality control was obtained through splicing, filtering, and clean tags. The operational taxonomic units (OTUs) of the clean tags were performed by UPARSE. Alpha and beta diversities were calculated using QIIME software and R software. Cluster analysis of the colonic content was proceeded by principal coordinate analysis (PCoA), and microflora were analyzed at phylum and family levels.

### Metabolomics analysis

According to a previous report, the metabolites in the colon tissue were extracted and measured by LC-MS [22]. The raw mass spectrum (MS) data was analyzed via Discoverer 3.1 (Thermo Fisher) to quantify metabolites and then analyzed by R package and MetaboAnalyst [23,24]. Principal component analysis (PCA) and supervised orthogonal partial least squares discriminant analysis (OPLS-DA) were performed. Cumulative R2 and Q2 were used to describe the quality and capability of the models obtained by PCA and OPLS-DA analysis. Variable importance in the projection (VIP) value and volcano plot were used to reveal pivotal metabolites. Metabolites with VIP >  1, P <  0.05, and fold change >  2 were accepted as diarrhea-associated biomarkers. Metabolites were identified by searching the MS/MS spectrum of precursor ions against the self-built database. Metabolic pathways annotation with Kyoto Encyclopedia of Genes and Genomes and MetaboAnalyst databases was performed.

### Statistical analysis

All numerical data was represented as mean ±  SD. Comparisons of statistical significance between the two rabbit groups were performed using Student’s t-test (for independent samples) via SPSS (16.0). Differences were considered to be significant at P < 0.05.

## Results

### Effects of FSAE in rabbits

**Fig 1:**
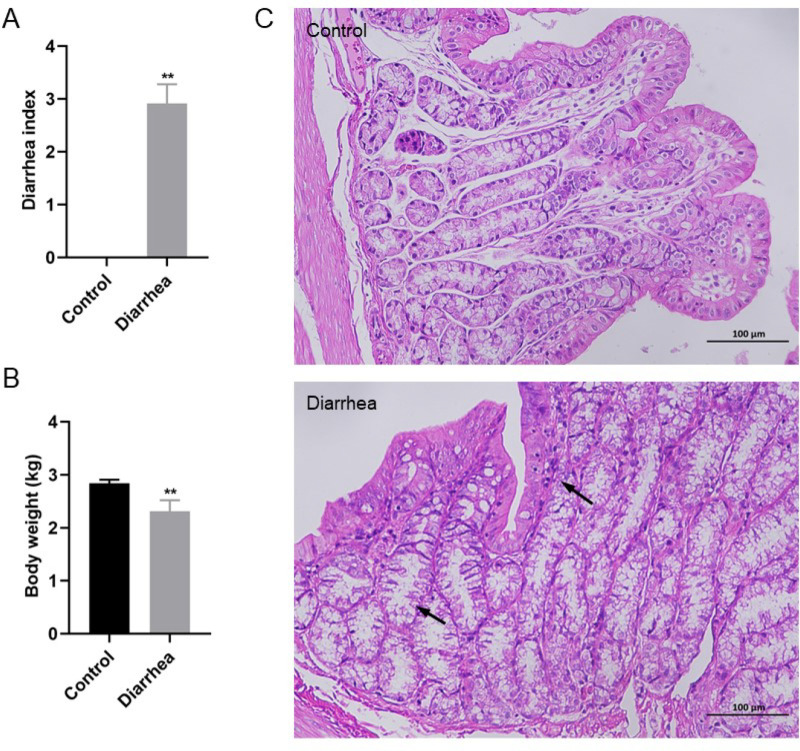
Effects of FSAE in rabbits (n =  6). A: diarrhea index; B: body weight; C: histological analysis of colon tissue. The arrows indicate inflammatory cell infiltration, necrosis, and epithelial destruction. Compared with the control group, ** P <  0.01 from the students’ t-test.

The diarrhea index and body weight were calculated (Fig 1A and B). The diarrhea index was significantly higher in FSAE group (2.91 ±  0.36) than the control group (0 ±  0) (P <  0.01). It was found that the rabbit body weight in the FSAE group (2.31 ±  0.31) was significantly lower compared to the control (2.84 ±  0.07, P <  0.01). As shown in Fig 1C, the diarrhea group exhibited pathological damages in the colon tissue compared with healthy animals with normal mucosal structure and epithelium, contrary to which inflammatory cellular infiltrates, necrosis, and disrupted epithelium (arrow pointing) were detected in diarrhea rabbits (Fig 1C).

### ELISA characterization

Ten indicators related to diarrhea in serum were investigated by ELISA (Table 1). The SS, SP, VaIP, CCK, and 5-HT levels were conspicuously increased in diarrhea animals (P <  0.01). However, the rabbits in the diarrhea group exhibited lower levels of GAS, MTL, ENK, and β-EP than those in the control group (P <  0.01). Regarding ACHE, there was no significant difference between the two animal groups (P >  0.05).

**Table 1:** ELISA results in serum indices between the control and diarrhea groups.

Variables	Control	Diarrhea
GAS	280 ± 30	193 ± 23**
MTL	1620 ± 127	951 ± 248**
SS	60 ± 11	118 ± 16**
SP	20 ± 8.5	66 ± 11**
VaIP	81 ± 24	179 ± 18**
CCK	129 ± 19	280 ± 35**
5-HT	34 ± 12	88 ± 8.0**
ACHE	118 ± 27	129 ± 35
ENK	37 ± 2.5	22 ± 3.9**
β-EP	45 ± 3	27 ± 5.2**

GAS: gastrin; MTL: motilin; SS: somatostatin; SP: Substance P; VaIP: Vasoactive intestinal peptide; CCK: Cholecystokinin; 5-HT: 5-hydroxytryptamine; ACHE: Acetylcholinesterase; ENK: enkephalin; β-EP: β-Endorphins. The unit of 5-HT and ENK was ng/mL; The unit of ACHE was nmol/mL; The unit of the other variable was pg/mL. Compared with the model group, ** P <  0.01 by Student’s t‑test.

### Colonic microbiota in rabbits with FSAE

**Fig 2:**
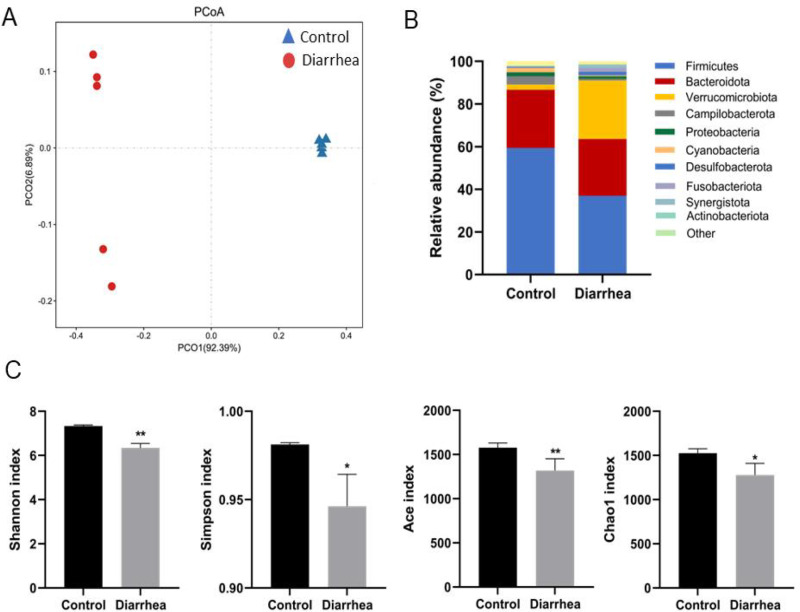
Effects of FSAE on gut microbiota in rabbit with diarrhea. A: PCoA plots of bacterial community structures in the colonic content of rabbits in the control and diarrhea groups. B: Relative abundances of the gut microbiotas at the phylum level in the control and diarrhea groups. C: Shannon, Simpson, Ace and chao1 index in the control and diarrhea groups. ** P <  0.01, *  P <  0.05 from the students’ t-test.

PCoA plot indicated a distinct cluster of the colonic content samples in different rabbit groups (Fig 2A). Indexes including Shannon, Simpson, ACE, and CHAO1 were significantly reduced in the rabbits with FSAE (Fig 2C). The relative abundance of colonic microbiota composition was shown in Fig 2B. Firmicutes, Campilobacterota, and Proteobacteria were significantly reduced whereas, Verrucomicrobiota was markedly increased in rabbits from diarrhea animals. According to statistical analysis, the important microbial genera were selected at the genus level (Table 2). Thirty-one remarkably different genera were found, with increased Akkermansia, Bacteroides, Phascolarctobacterium, Rikenella, Fusobacterium Lachnoclostridium, and Bilophila*,* and decreased Ruminococcus, Campylobacter and Roseburia (P <  0.01).

**Table 2:** Relative abundance of microbiota in colonic content at the genus level (n =  5).

Genus	Control	Diarrhea	Fold Change
*Akkermansia*	2.42 ± 0.15	27.39 ± 7.26**	11.30
*Bacteroides*	2.91 ± 0.19	13.13 ± 5.61**	4.52
*Ruminococcus*	5.11 ± 0.29	1.04 ± 0.25**	0.20
*V9D2013_group*	4.64 ± 0.14	1.12 ± 0.13**	0.24
*Phascolarctobacterium*	0.72 ± 0.07	3.9 ± 0.21**	5.45
*Campylobacter*	3.83 ± 0.21	0.75 ± 0.26**	0.20
*Rikenella*	0.25 ± 0.03	3.65 ± 1.91**	14.49
*Eubacterium_ruminantium_group*	3.00 ± 0.08	0.36 ± 0.07**	0.12
*NK4A214_group*	1.25 ± 0.21	2.05 ± 0.38**	1.64
*Roseburia*	2.14 ± 0.31	0.50 ± 0.05**	0.23
*Parabacteroides*	0.93 ± 0.06	1.58 ± 0.32**	1.70
*Alistipes*	1.53 ± 0.11	0.69 ± 0.35**	0.45
*Fusobacterium*	0.06 ± 0.02	1.74 ± 0.18**	28.97
*Colidextribacter*	0.67 ± 0.06	1.05 ± 0.16**	1.56
*dgA-11_gut_group*	1.52 ± 0.11	0.18 ± 0.04**	0.12
*Desulfovibrio*	0.31 ± 0.03	1.18 ± 0.49**	3.81
*Synergistes*	0.12 ± 0.03	1.31 ± 0.26**	10.64
*UCG-005*	0.74 ± 0.05	0.36 ± 0.07**	0.48
*Papillibacter*	0.29 ± 0.04	0.48 ± 0.07**	1.66
*Eubacterium_siraeum_group*	0.53 ± 0.05	0.15 ± 0.06**	0.28
*Intestinimonas*	0.03 ± 0.01	0.57 ± 0.08**	17.21
*Butyricimonas*	0.09 ± 0.02	0.5 ± 0.16**	5.33
*Oscillibacter*	0.05 ± 0.01	0.46 ± 0.05**	9.79
*Lachnoclostridium*	0.03 ± 0.02	0.31 ± 0.11**	11.82
*Bilophila*	0.02 ± 0.01	0.31 ± 0.1**	18.59
*Escherichia-Shigella*	0.03 ± 0.02	0.18 ± 0.09**	6.51
*Anaerovorax*	0.05 ± 0.01	0.12 ± 0.02**	2.33
*Monoglobus*	0.08 ± 0.02	0.03 ± 0.01**	0.43
*Cloacibacillus*	0.01 ± 0.01	0.08 ± 0.02**	9.80
*UCG-008*	0.00 ± 0.00	0.08 ± 0.02**	18.73
*Methanobrevibacter*	0.08 ± 0.03	0.00 ± 0.00**	0.05

Fold change: diarrhea group vs control group; Compared with the model group, ^**^ *P* <  0.01 by Student’s t‑test.

### Colon tissue metabolic profiling

**Fig 3:**
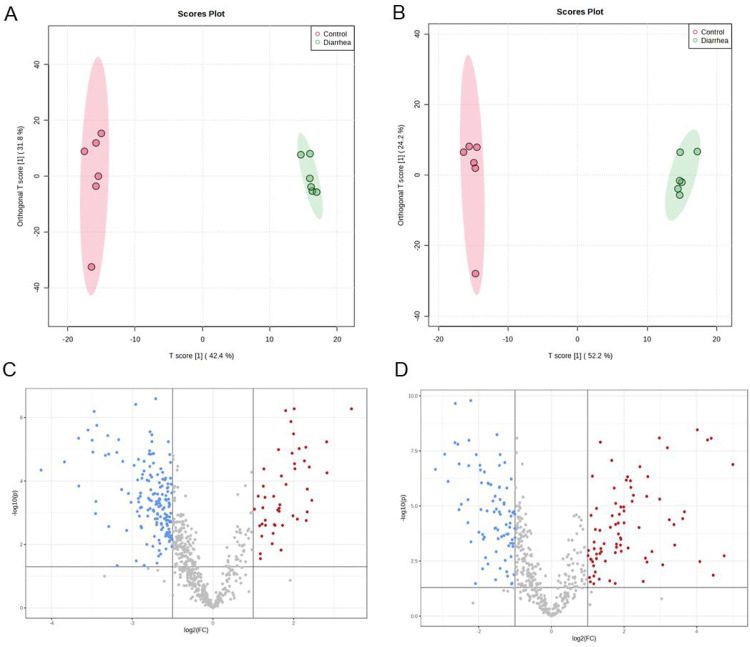
OPLS-DA score plot and volcano plot in the control and diarrhea groups. A and B: OPLS-DA score plots in the positive and negative mode, respectively. C and D, volcano plot of the metabolites in the control and diarrhea groups, FC >  2 and P <  0.05.

The metabolomics method was applied to investigate the changes in the metabolites in the colon of rabbits. The unsupervised PCA analysis examined the metabolic profiling in colon tissue in both positive and negative modes. The four QC samples were tightly clustered, suggesting that the robustness of the analytical method is stable and repeatable. Moreover, a slight separation trend of the samples in the control and diarrhea groups was found in the PCA score plots, which indicated changes in metabolic profiling in the colon tissue of the rabbits with FSAE treatment. Supervised OPLS-DA was performed to identify the biomarkers. The score plots of OPLS-DA are shown in Fig 3A and 3B; the OPLS-DA score plots showed that the diarrhea animals were far away from healthy rabbits.

### Metabolites related to diarrhea and pathway analysis

In Fig 3C and 3D, 209 and 164 different metabolites were found in the positive and negative ion modes, respectively. Given the VIP value obtained from the OPLS-DA models, 13 metabolites were identified as the most important metabolites associated with diarrhea (Table 3). Compared to the control group, levels of 6 metabolites such as elaidic acid, adrenic acid, glucose-6-phosphate, docosahexaenoic acid, oleamide, and stearoyl ethanolamide were signally increased (P <  0.01), and 7 metabolites including citric acid, taurine, glucose, acetyl-L-carnitine, glutamine, betaine, and carnosine were dramatically decreased in the diarrhea rabbits (P <  0.01).

**Table 3:** Potential metabolites in the colonic tissue related with diarrhea induced by FSE.

Metabolites	Formula	RT	m/z	Fold Change	VIP
Elaidic acid	C_18_H_34_O_2_	10.44	281.2484	2.40**	1.16
Adrenic acid	C_22_H_36_O_2_	10.45	331.2642	4.24**	1.25
Citric acid	C_6_H_8_O_7_	2.04	145.0141	0.45**	1.23
Glucose-6-phosphate	C_6_ H_13_O_9_P	1.33	259.0223	7.30**	1.12
Taurine	C_2_ H_7_NO_3_S	1.29	124.0073	0.40**	1.30
Docosahexaenoic Acid	C_22_H_32_O_2_	9.72	327.2329	2.25**	1.26
Glucose	C_6_H_12_O_6_	1.37	225.0618	0.36**	1.28
Oleamide	C_18_H_35_NO	9.81	282.279	2.18**	1.17
Stearoyl Ethanolamide	C_20_H_41_NO_2_	10.56	310.3104	2.66**	1.31
Acetyl-L-carnitine	C_9_H_17_NO_4_	1.79	204.1231	0.32**	1.38
Glutamine	C_5_H_10_N_2_O_3_	1.29	147.0765	0.48**	1.19
Betaine	C_5_H_11_NO_2_	1.32	118.0865	0.27**	1.33
Carnosine	C_9_H_14_N_4_O_3_	1.26	227.1139	0.33**	1.31

RT: retention time; Fold change: the diarrhea group compared with the control group; ** P <  0.01 as compared with the control group from Student’s t-test; VIP: variable importance in projection from OPLS-DA models.

**Fig 4:**
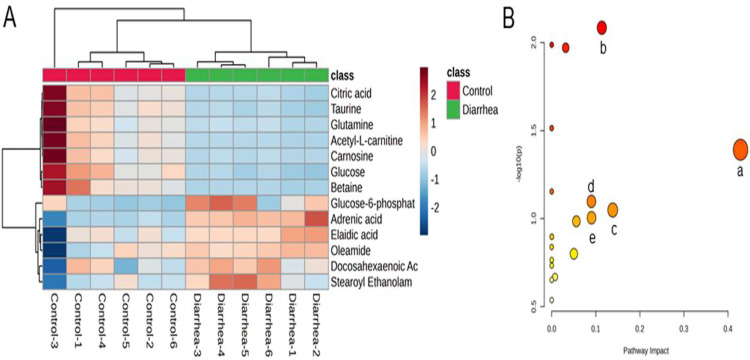
Heatmap and pathway analysis of the potential metabolites in colon tissue related with diarrhea. A: Hierarchical clustering analysis of differential metabolites. Red color: indicating high abundance, blue color indicating low abundance. B: Disturbed pathways related with the metabolites in colon tissue. a: Taurine and hypotaurine metabolism; b: Alanine, aspartate and glutamate metabolism; c: Starch and sucrose metabolism; d: Histidine metabolism; e: Citrate cycle (TCA cycle).

The identified metabolites are indicated by heatmap to display the differences among colon tissue samples intuitively. The relative intensity of the vital metabolites varied between the two groups as shown by the red and blue colors in Fig 4A. Furthermore, the samples representing diarrhea animals were clustered and separated from control rabbits in the vertical axis of the dendrogram by hierarchical cluster analysis (Fig 4A). The affected metabolic pathways related to diarrhea in rabbits were identified with metabolite pathway analysis. As shown in Fig 4B, results reveal that taurine and hypotaurine, slanine, aspartate and glutamate, starch and sucrose, histidine and citrate cycle metabolisms were associated with the different metabolites induced by FSAE in rabbits.

## Discussion

*Folium Sennae* is a commonly used Chinese herbal medicine for treating constipation and is also used in research to establish experimental diarrhea animal models [11]. In this study, rabbits were used to investigate the pathogenesis of diarrhea induced by FSAE. Results revealed that the diarrhea index of rabbits in the diarrhea group increased significantly, whereas, body weight decreased significantly. Diarrhea is often accompanied by intestinal damage, and results of histopathological examinations revealed that the rabbits in the diarrhea group had significantly higher inflammatory cell infiltration compared to rabbits in the control group.

Gastrointestinal peptides are produced and secreted by intestinal endocrine cells. These peptides play regulatory roles in different physiological functions of the digestive system. Among these peptides GAS can regulate gastric acid secretion, nourish gastrointestinal mucosa, and regulate immune cells [25]. The MTL can activate gastrointestinal smooth muscle, promote gastrointestinal motility, accelerate gastric emptying, and promote insulin secretion [26]. The SS can inhibit the release of GAS, inhibit gastric acid and gastric peristalsis production, and relax blood smooth muscle. The SP can cause vasodilation and increased vascular permeability of the intestine [27]. Vasoactive intestinal peptide (VIP) can relax blood vessels, regulate gastric and intestinal smooth muscle relaxation, stimulate gland secretion, and inhibit gastric acid secretion. Cholecystokinin (CCK) is a gastrin-related peptide that digests food and absorbs nutrients by stimulating gallbladder contraction, as well as pancreatic exocrine secretion and inhibiting gastric emptying. In this study, the contents of GAS and MTL decreased significantly whereas, SS, SP, VIP, and CCK increased significantly, indicating gastrointestinal dysfunction as one of the major factors causing diarrhea in rabbits.

The enteric nervous system releases different neurotransmitters to regulate intestinal motility. 5-HT is involved in gastrointestinal motility and secretion, and it can stimulate Ca2 + signals in the mucosal epithelium and intestinal neurons. Its abnormal expression can lead to many diseases. As the intestinal cells of rabbits in the diarrhea group were damaged, higher expression of 5-HT was observed [28]. Other neurotransmitters, such as Enkephalin (ENK) and β-Endorphins (β-EP) have vital regulatory roles in gastrointestinal signaling, resulting in altered fluid and electrolyte movement [29].

Diarrhea results from intestinal flora imbalance [30,31]. The 16S rDNA technology exhibited a changed composition of intestinal flora in diarrhea rabbits. The ratio of Firmicutes/Bacteroidota in the diarrhea group was significantly decreased whereas, the abundance of Verrucomicrobia was significantly increased. The ratio of Firmicutes/Bacteroidota is associated with dysbacteriosis [32–34]. Alpha diversity was significantly changed in the diarrheal animals. These observations suggested that diarrhea induced by FSAE is due to the changes in the diversity and composition of intestinal flora.

Gut microbiota constitutes a “second genome” in humans, which is characterized by its vast numbers, diverse populations, and intricate classification of microbes. The healthy or diseased status of an animal is intertwined with gut microbiota balance. This equilibrium is maintained through factors like quorum sensing, nutrient competition, and reciprocal modulation of metabolites. Gut microbiota remains relatively stable, ensuring the overall health of the host. However, when the host is exposed to external stimuli, disruptions in the gut flora can occur, which increases the host’s susceptibility to many diseases. Connections between gut microbiota metabolic dysregulation and diarrhea have been studied to identify specific bacteria associated with diarrhea [35,36].

By analyzing differential microbial communities between two groups the current study observed notable changes in the colonic microbiota of rabbits fed FSAE. Specifically, the abundances of bacteria like Akkermansia, Bacteroides, Fusobacterium, and Bilophila were significantly high whereas, species such as Ruminococcus, Campylobacter, Alistipes, and Monoglobus were significantly low. Our results are similar to Li et al. [12]. Where colitis-prone mice exhibited elevated levels of bacteria like Bacteroides, Escherichia Shigella, and Desulfovibrio, this study further confirmed that these bacteria were related to diarrhea in rabbits induced by FSAE. The decline in beneficial bacteria, such as Ruminococcus, is particularly significant. This bacterium species is recognized for its role in fiber degradation and the generation of short-chain fatty acids (SCFA), which are essential for preserving gut health and mitigating inflammation [37]. A reduction in Ruminococcus results in diminished SCFA production and compromised digestion of complex carbohydrates, hence weakening the mucosal barrier and intensifying intestinal inflammation [38]. Therefore, addressing this imbalance via the use of probiotics, prebiotics, and feed additives may boost the proliferation of SCFA-producing bacteria, representing a possible therapeutic approach for controlling diarrhea resulting from FSAE-induced dysbiosis.

The elevation of bacteria such as Fusobacterium and Bilophila also correlates with the occurrence of diarrhea. Fusobacterium is an opportunistic pathogen that produces virulence factors like lipopolysaccharides (LPS), endotoxins, and hemolysins, contributing to intestinal inflammation. Similarly, Bilophila is also an opportunistic pathogen with strong associations with intestinal inflammation and gut barrier dysfunction [39–41]. Other bacteria with increased abundances due to FSAE treatment are often implicated in intestinal pathogenesis, facilitating the development of diarrhea. Eubacterium and Roseburia are components of a healthy colon and can produce short-chain fatty acids with anti-inflammatory functions and protect mucosal barrier integrity [42,43]. However, their abundances were significantly decreased in the colon of rabbits induced with diarrhea. Alistipes exhibited significantly reduced abundance in previous research [44], while this genus has been linked to colon-related diseases like colorectal cancer associated with anti-inflammatory and gut-protective effects. This discrepancy stems from specific species within the Alistipes genus. In this study, a reduction in the abundance of Alistipes in the rabbit diarrheal model is attributed to the decline in beneficial bacterial species. Dysbiosis of the gut microbiota is marked by an increase in pathogenic bacteria and a decrease in useful bacteria by FSAE-induced diarrhea.

Metabolomics can analyze metabolic traits and has been extensively used to reveal disease development mechanisms. The involvement of metabolites like amino acids, bile acids, and lipids has been reported in the progression of diarrhea [45,46]. Studies on diarrhea primarily focus on detecting metabolites in gut contents, urine, and blood, with a limited investigation of the colon. Colon is associated with diarrhea, which exhibits metabolic changes intimately linked to intestinal structure. The current study conducted positive and negative ion partial PLS-DA on the detected substances. Results revealed separated patterns in different rabbits, indicating alterations in induced metabolic pathways by FSAE. Furthermore, the study identified 13 significantly altered differential metabolites (such as Elaidic acid, Adrenic acid, and Glucose-6-phosphate) participating in different pathways observed only in rabbits with diarrhea.

Elaidic acid is a trans-fatty acid that induces oxidative stress, endothelial injury, and systemic inflammation [47]. In this study, elaidic acid levels in the diarrhea-modeled group were 2.4 times higher compared to the control group, suggesting its potential involvement in colon tissue damage. Significantly elevated levels of docosahexaenoic acid in rabbits were observed in this study, which is in line with the findings by Arnold et al. [48]. Furthermore, substances like betaine, taurine, and carnosine were significantly downregulated in animals with diarrhea. Betaine possesses anti-tumor, anti-ulcer, and gastrointestinal regulatory effects [49]. In this study, the betaine content in animals with diarrhea decreased by 0.27 times compared to the control group. A previous study reported that adding betaine to piglets’ diet reduced diarrhea incidence by enhancing intestinal barrier integrity, tight junction protein expression, and antioxidant activity [50]. Citric acid is a potent organic acid for maintaining pH balance, and it stimulates macrophages to secrete cytokines promoting B lymphocyte differentiation, enhances serum IgA and IgG levels for immune function, and suppresses inflammatory factors to improve gut barrier integrity, ultimately lowering diarrhea rates [51]. In this study, colon tissue citric acid content was significantly decreased by FSAE, which negatively impacts gut health.

The importance of glutamine in treating diarrhea across various species has been studied recently [52], and it has been suggested that dietary glutamine supplementation aids in repairing damaged intestinal barriers and boosting gut immunity ameliorating diarrhea [53–55]. Therefore, the reduced glutamine levels observed in this study contribute significantly to diarrhea caused by Senna leaves. Furthermore, other decreased substances like Carnosine and Taurine were found to be negatively correlated with diarrhea [56,57]. Supplementation of Senna leaves led to alterations in metabolites within rabbit colon tissue, increasing unfavorable diarrhea-related substances while reducing anti-inflammatory and gut-maintaining substances, ultimately contributing to diarrhea onset.

The present study highlights that FSAE induces diarrhea in rabbits by altering the intestinal microbiota, disrupting metabolic pathways, and dysbiosis of gastrointestinal peptides. Our results are consistent with previous studies that reported that plant-based laxatives may disrupt gut physiology, malabsorption, and dysbiosis, leading to diarrhea [58,59]. These results suggest that diarrhea in livestock can be controlled through the targeted application of probiotics, prebiotics, and feed additives to restore microbial balance and enhance intestinal health. Furthermore, optimizing feed formulations to modulate gastrointestinal peptides offers potential therapeutic approaches to improve animal health and productivity [60].

## Conclusion

Diarrhea is a major health problem in animal production, leading to reduced growth rates, inefficient feed efficiency, and increased mortality. Our results revealed that rabbits treated with FSAE had reduced body weight, higher diarrhea index, and significant alterations in gastrointestinal peptides, microbial diversity, and metabolic pathways. Moreover, we concluded that diarrhea induced by FSAE is associated with gut microbiota dysbiosis and changes in metabolomic pathways in the intestine. Our findings offer a novel model for investigating GIT dysfunction and its potential treatments.
